# Structural insights into the binding of bS1 to the ribosome

**DOI:** 10.1093/nar/gkad126

**Published:** 2023-02-25

**Authors:** Gaetano D’Urso, Sophie Chat, Reynald Gillet, Emmanuel Giudice

**Affiliations:** Univ. Rennes, CNRS, Institut de Génétique et Développement de Rennes (IGDR) UMR6290, 35000 Rennes, France; Univ. Rennes, CNRS, Institut de Génétique et Développement de Rennes (IGDR) UMR6290, 35000 Rennes, France; Univ. Rennes, CNRS, Institut de Génétique et Développement de Rennes (IGDR) UMR6290, 35000 Rennes, France; Univ. Rennes, CNRS, Institut de Génétique et Développement de Rennes (IGDR) UMR6290, 35000 Rennes, France

## Abstract

The multidomain ribosomal protein bS1 is the biggest and the most flexible and dynamic protein in the 30S small subunit. Despite being essential for mRNA recruitment and its primary role in the accommodation of the start codon within the decoding centre, there has not yet been a high-resolution description of its structure. Here, we present a 3D atomic model of OB1 and OB2, bS1’s first two N-terminal domains, bound to an elongation-competent 70S ribosome. Our structure reveals that, as previously reported, bS1 is anchored both by a π-stacking to the 30S subunit and via a salt bridge with the Zn^2+^ pocket of bS1. These contacts are further stabilized by other interactions with additional residues on OB1. Our model also shows a new conformation of OB2, interacting with the Shine–Dalgarno portion of the mRNA. This study confirms that OB1 plays an anchoring role, but also highlights a novel function for OB2, which is directly involved in the modulation and support of mRNA binding and accommodation on the ribosome.

## INTRODUCTION

The flow of genetic information from DNA to functional proteins is achieved through the translation of mRNA molecules by ribosomes. The initiation of this translation process is the rate-limiting step for protein synthesis (1). In prokaryotes, the recognition of the first codon by P-site fMet-tRNA^fMet^ relies on mRNA binding to the small ribosomal subunit, 30S (2). This process is driven by the interaction between the AG-rich ribosome-binding site of the mRNA’s 5′ untranslated region (5′ UTR) known as the Shine–Dalgarno (SD) sequence (3) and its counterpart, the CU-rich anti-SD (aSD) sequence at the 3′ end of 16S rRNA (4). The duplex formed by the two RNA molecules proceeds through various base-pairing interactions, ensuring that the 30S initiation complex is formed in such a way that the ‘start’ codon of the open reading frame (usually an AUG) is correctly placed at the P site (5,6). This interaction between the SD and the aSD is sufficient for most mRNAs to initiate translation. However, some natural mRNAs have weak SD sequences, none at all or structural motifs in the 5′ UTR upstream from the coding portion, and these will all impede interactions with the 16S rRNA (7–9). In such cases, other factors are required to form the translation initiation complex.

The ribosomal protein bS1 is a single-stranded RNA-binding protein that is conserved in all Gram-negative bacteria. More distant ‘S1 proteins’, generally formed of fewer domains, have also been identified in Gram-positive bacteria (10). With a length in solution of ∼230 Å (11), it is the largest and most acidic protein that interacts, although in a weak and reversible way, with the small subunits of bacterial ribosomes (12). It is instrumental not just during the late steps of canonical translation initiation, but also in facilitating the binding of the small subunit to the mRNA SD portion (13,14). The structure of bS1 contains six consecutive domains connected by loops of 10–15 residues that provide the flexibility essential for accomplishing its principal function, that of recruiting mRNA transcripts on the ribosome (15,16). Each bS1 domain, or oligonucleotide/oligosaccharide-binding (OB)-fold domain, consists of ∼70 amino acids folded into a β-barrel. The barrel is composed of five antiparallel β-strands arranged into a Greek key topology and one α-helix, which closes the bottom (15). From a functional point of view, the first two N-terminal domains (OB1 and OB2) do not have detectable RNA-binding activity, and they seem to be mostly involved in binding ribosomes (10,15–20). More specifically, the protein’s first 106 amino acids are sufficient to ensure the contact with the 30S subunit by means of an interaction with the protein uS2 (10,15–21). However, because OB2 is located near the 16S helix h26 and the 5′ end of mRNA, it has also been suggested that it is involved in mRNA interaction on the ribosome (20). In contrast, the bS1 C-terminal portion (formed by domains OB3–OB6) is essential for RNA binding (13,16,22–24).

Besides its primary role in translation initiation, the bS1 protein is also involved in many other important cellular mechanisms. For instance, bS1, EF-Tu and EF-Ts are the essential host-derived subunits of Q-Beta replicase (QBR), an enzyme that directly replicates RNA from the genomic RNA positive strand in *Escherichia coli* (25–27). The OB1 and OB2 domains are essential for establishing interactions between these three protein partners during formation of the QBR, while OB3 is required for the efficient recognition and synthesis of the negative-strand RNA (25–27). In addition, bS1 is a potent activator of transcriptional cycling, affecting the transcriptional activity of the RNA polymerase (28). The protein is also involved in *trans*-translation, where it binds tmRNA (29–33), as well as intervening to protect single-stranded RNAs from degradation by RNase E (34).

**Figure 1.**
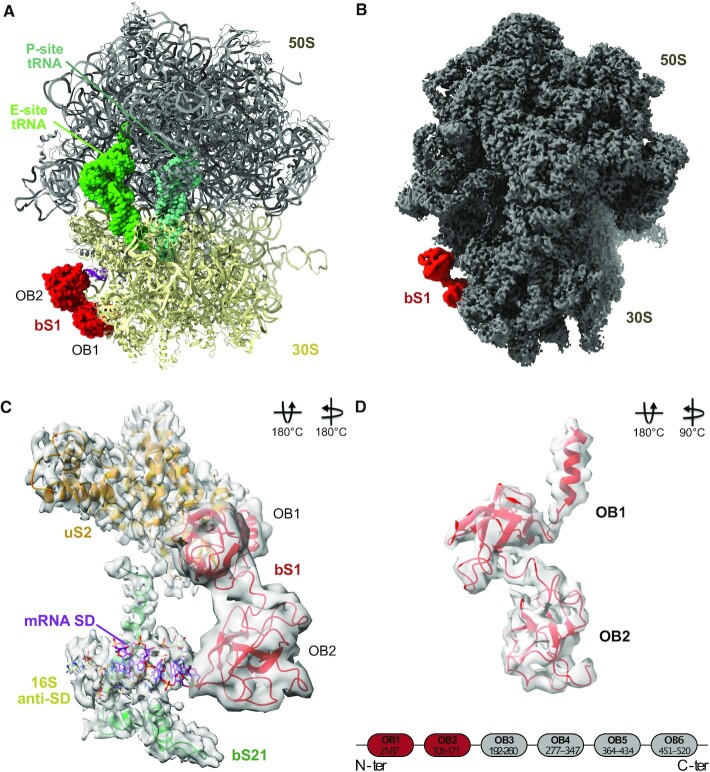
Structure of the EC ribosome in complex with bS1. (**A**) Atomic model of the translating *E. coli* ribosome in complex with the first two N-terminal domains of bS1, OB1 and OB2. The 50S subunit is grey, 30S is gold, P-site tRNA is aquamarine, E-site tRNA is green, the mRNA SD sequence is purple and bS1 is red. (**B**) Cryo-EM density map of the 70S ribosome. bS1 is in red. (**C**) Focus on the bS1 binding site, with uS2 orange, 16S aSD khaki, bS21 aquamarine, SD purple and bS1 red. (**D**) Secondary structure and atomic model of OB1 and OB2. In the secondary structure, the domains present in our model are highlighted in red.

Although the bS1 protein is obviously of primary importance, there has been no high-resolution structure, a description that is essential to better understand its participation in various molecular mechanisms. To address this, we present here a cryogenic electron microscopy (cryo-EM) structure at an overall resolution of 3.42 Å, showing the N-terminal portion of bS1 interacting with a 70S elongation-competent (70S EC) complex in the presence of an mRNA carrying the SD six-base consensus sequence AGGAGG. This new atomic model reveals a previously unseen conformation of the protein, with the OB2 domain interacting with the SD portion of the mRNA. This highlights the fact that OB2 has an RNA-binding role when bS1 is already bound to the 30S subunit (Figure 1). Our study provides new insights into the role of bS1’s N-terminal domains during the initial steps of translation, improving our knowledge of the mechanisms underlying the protein's functions.

## MATERIALS AND METHODS

### Ribosome purification

Ribosomes were purified from *E. coli* MG1655. When the culture reached an OD_600_ of 0.8, cells were pelleted, resuspended in FP buffer (20 mM Tris–HCl, pH 7.5, 50 mM MgOAc, 100 mM NH_4_Cl, 0.5 mM EDTA and 1 mM DTT) and lysed in a French press. The lysate was then clarified by centrifugation at 20 000 × *g* for 45 min at 4°C. Next, the supernatant was layered 1:1 (v/v) over a high-salt sucrose cushion buffer (10 mM Tris–HCl, pH 7.5, 10 mM MgOAc, 500 mM NH_4_Cl, 0.5 mM EDTA, 1.1 M sucrose and 1 mM DTT). After ultracentrifugation at 92 000 × *g* for 20 h at 4°C, the resulting ribosome pellets were resuspended in 1 ml of ‘Ribo_A’ buffer (10 mM Tris–HCl, pH 7.5, 10 mM MgCl_2_, 50 mM NH_4_Cl, 0.5 mM EDTA and 1 mM DTT). To isolate the 70S ribosomes from 30S and 50S ribosomal subunits, the ribosomes were centrifuged at 95 000 × *g* for 18 h at 4°C through a 10–45% (w/w) linear sucrose gradient in Ribo_A buffer. Gradients were fractionated before determining the *A*_260_ absorbance profiles. Fractions corresponding to the 70S peak were mixed and diluted in Ribo_A buffer for a final ultracentrifugation at 92 000 × *g* for 20 h at 4°C. The ribosomal pellets were resuspended in Ribo_A buffer, and flash frozen and stored at −80°C.

### Sample preparation and cryo-EM data collection

To prepare the 70S EC complex, 25 pmol of tRNA^fMet^ (ICNA0219915410; VWR) was first refolded for 2 min at 80°C in ‘Buffer I’ (10 mM HEPES–KOH, pH 7.5, 25 mM MgCl_2_ and 20 mM NH_4_Cl). This was followed by a second incubation at room temperature for 30 min. Purified 70S ribosomes (12.5 pmol) were incubated for 20 min at 37°C in ‘Buffer III’ (10 mM MgOAc, 10 mM NH_4_Cl, 50 mM KCl, 5 mM HEPES–KOH, pH 7.5, and 1 mM DTT) with 25 pmol of a truncated version of cspA mRNA (35) in which the 5′ UTR was completely deleted to avoid any interference with the ribosome and 25 pmol of the folded tRNA^fMet^. The cspA mRNA fragment contained the SD sequence and a linker to place the AUG codon into the P site: 5′-GGGCUUAAGUAU**AAGGAGG**AAAAAAUAUGCCACAGGGAACUGUGAAGUGGUUCAACGCGGAGAAGGGGUUCGGCUUUAUCGCCCCCGAAGACGGUUCCGCGGAUGUAUUUGUCCACUACACGGAGAUCCAGGGAACGGGCUUCCGCACCCUUGAAGAAAACCAGAAGGUCGAGUUCGAGAUCGGCCACAGCCCUAAGGGCCCCCAGGCCACCGGAGUCCGCUCGCUCUGA-3′ (the SD sequence is given in bold and the methionine start codon is underlined). To confirm that the positioning of S1 is not dependent on this specific sequence, we also performed data analysis using another synthetic mRNA previously used to study *trans*-translation (36) with the sequence 5′-**AGGAGGU**GAGGUUUU-3′ (the SD sequence is given in bold and the P-site phenylalanine codon is underlined). After adjusting concentrations to 160 nM in Buffer III, samples were directly applied to glow-discharged holey carbon films (Quantifoil 3.5/1). These grids were flash frozen in liquid ethane using an FEI Vitrobot Mark III. They were then imaged at the IECB Structural Biophysical Chemistry Platform using a 200 kV Talos Arctica cryo-TEM (Thermo Fisher Scientific) equipped with a field emission gun. SerialEM software was used to automatically record 6381 movies under low-dose conditions with a Gatan K2 direct electron detector with a defocus range of 0.4–2.0 μm and a pixel size of 0.9291 Å.

### Image processing

The initial steps of single particle analysis were done using cryoSPARC (version 2.15.0) (37). Movies were motion corrected using Patch Motion, and Patch CTF was used for contrast transfer function (CTF) estimation. Particles were manually picked from 18 micrographs using cryoSPARC. To generate an initial model for template picking, the resulting 476 particles were subjected to a first run of 2D classification into eight distinct classes. Of these, six classes (a total of 401 particles) were selected and used as templates to pick particles in the entire dataset of 6381 micrographs. The selected particles were inspected, extracted from the micrographs and then underwent two rounds of 2D classification (200 classes each). We retained 294 820 particles that were used to generate an initial *ab initio* model, and then refined with the legacy version of homogeneous refinement in cryoSPARC. The micrographs and particle coordinates were transferred to RELION (version 3.1 beta) (38). Movies were once again corrected for the effects of drift and beam-induced motion using MotionCor2 software (39). CTF parameters were re-estimated using Gctf software (40). A first 3D auto-refinement was performed to reconstruct a density map of the ribosome using the cryoSPARC homogeneous refinement map as an initial model. The heterogeneity of the dataset was then assessed using a 3D classification into 12 classes, which separates the 50S subunit (Classes 10 and 12), junk particles (Class 8), ribosomes in ratcheted conformations (Classes 7 and 11) and canonical ribosomes with feeble and noisy densities attributed to the bS1 protein (Classes 1, 2, 3, 4, 5, 6 and 9; 175 859 particles). Overall, this unbiased and unfocused classification process did not uncover a well-resolved conformation for bS1, but it did allow us to minimize ribosomal heterogeneity resulting from different subunit conformations as well as filtering out ‘junk particles’ from the entire dataset. To further focus on bS1 and limit the classification to the ribosomal portions known to interact with that protein, we used UCSF Chimera (41) to create a spherical mask with a radius of 50 Å. We then subtracted the ribosomal signal and performed a 3D classification with six classes, without alignment on the region of interest. The particles corresponding to bS1 were selected (Classes 1, 3, 4 and 6; 141 003 particles), reverted to the original one and subjected to a second round of 3D classification with signal subtraction and using a tighter spherical mask of 40 Å. The dataset was split into six classes, and Class 5 (containing 33 572 particles) was selected and reverted to the original images. After refinement, the resulting map still displayed heterogeneity. To further limit this, a final 3D classification (three classes) was done with the 70S signals removed using a mask created with the Segger volume data partition extension (42) of Chimera. From the resulting classification, we selected 16 381 particles (Class 3). The dataset was reverted to the original and 3D refined. The particles were then CTF refined and polished. Finally, we reconstructed the map, which was then post-processed with a solvent mask to produce an overall resolution of 3.42 Å (Supplementary Figures S1 and S2).

### Model reconstruction

To build and refine an atomic model of bS1, the high-resolution cryo-EM map was further processed using the CCP-EM software suite (version 1.5.0) (43). First, the small bS1 density was isolated from the entire map using the Segger tool of Chimera. The protein coordinates were extracted from the 6BU8 PDB file (20) and rigid body fitted into the bS1 volume. The map was converted into the MTZ format, and the model underwent two consecutive RIBFIND runs (44) to identify rigid-body elements after calculating the spatial proximities between the protein’s secondary structures. To optimize the fit to the experimental volume, the previously calculated rigid-body domain information was entered into Flex-EM (45). The model was then refined against the electron density with REFMAC5 software (46) using its jelly-body restraints option. The resulting density map showed regions at different resolutions—mostly because the images are centred on the ribosome, so peripheral zones are blurry, but also because of the higher flexibility of certain molecular components. These factors lead to a falloff in amplitude, resulting in the poor density contrast that is typical of the most dynamic portions of molecular complexes. We used LocScale (47) to compensate for the loss of information on the protein, enhance the interpretability of the bS1 volume and facilitate the fitting of the atomic model. This program uses prior information from the refined bS1 coordinates to improve the contrast of cryo-EM maps, and we downweighted the ribosomal signal in order to focus on the zone corresponding to bS1. The entire map was then used to rigid body fit the atomic model of the complex in Chimera, and the atomic model was manually adjusted in Coot (48). A final round of PHENIX (version 1.18.2) (49) real-space refinement (50) allowed us to improve the fitting of the coordinates against the sharpened volume. Once the model was refined, atomic model geometry, density fit and FSC curves were calculated using the PHENIX cryo-EM comprehensive validation tool. All figures shown here were created with Chimera.

## RESULTS

### bS1 forms a highly dynamic complex with the ribosome

The protein bS1 is the ‘mRNA-catching arm’ of the ribosome. Formed by six highly flexible OB-fold domains, and attached to the ribosomal small subunit through its N-terminal domain, bS1 acts as a dynamic mesh to modulate the binding, folding and movement of mRNA (20). While the protein’s activity is crucial for translation initiation, its high flexibility makes it a challenging subject for structural biology. For our investigations, we therefore used image analysis protocols to focus on a particular region of a big complex, such as the ribosome (see the ‘Materials and Methods’ section). This targeted approach has proven to be quite successful for separating different ribosomal conformations and for improving model resolution, especially when it comes to highly dynamic regions (51). Our structure consists of a 70S ribosome in complex with a short mRNA (see the ‘Materials and Methods’ section for sequence), a P-site tRNA, an E-site tRNA and the first two domains of bS1, OB1 and OB2 (Figure 1). Consistent with previous reports, bS1 binds at the mRNA exit channel, in the narrow cleft between the 30S head and platform (Figure 1A and B). The mRNA exit channel is formed by uS2, bS18, bS21 and the 3′ end of the 16S rRNA needed for the stabilization of the mRNA through the SD–aSD base pairing (Figure 1C). The mRNA contains the SD portion at its 5′ end, and is properly paired with the 16S aSD sequence. The tRNA in the P site is paired with the AUG starting codon, and this interaction is essential for the correct formation of the complex. Our complex looks therefore like a 70S EC (51), although a second tRNA is observed in the E site, presumably due to the excess of tRNA used during the *in vitro* complex formation. Translation initiation is driven and controlled by the three initiation factors IF1, IF2 and IF3. These ensure that the 70S initiation complex matures into a 70S EC complex whose P site contains a tRNA^fMet^ properly paired with the mRNA start codon (52). During translation initiation, bS1 facilitates the recruitment and correct positioning of the mRNA, giving life to a ribosome that is ready to translate (19). This description fits well with the present structure, which contains bS1 still bound to the small subunit of a 70S EC. Only the first two bS1 N-terminal domains, the most stable portions of the protein, were sufficiently resolved to allow reconstruction (Figure 1D), as the rest of the protein elements are extremely flexible and dynamic. It has to be noted that the same structure was also observed when using a different mRNA deprived of nucleotides upstream from the SD (sequence 5′-**AGGAGGU**GAGGUUUU-3′ with the SD sequence given in bold and a P-site phenylalanine codon underlined) (Supplementary Figure S3). This suggests the importance of the SD **AGGAGG** sequence in the positioning of S1. Therefore, this new model allows us to understand how these two bS1 domains interact with the 70S EC and help bring and stabilize the mRNAs waiting to be translated.

### bS1 requires unique interactions to anchor the small subunit of the 70S EC

**Figure 2.**
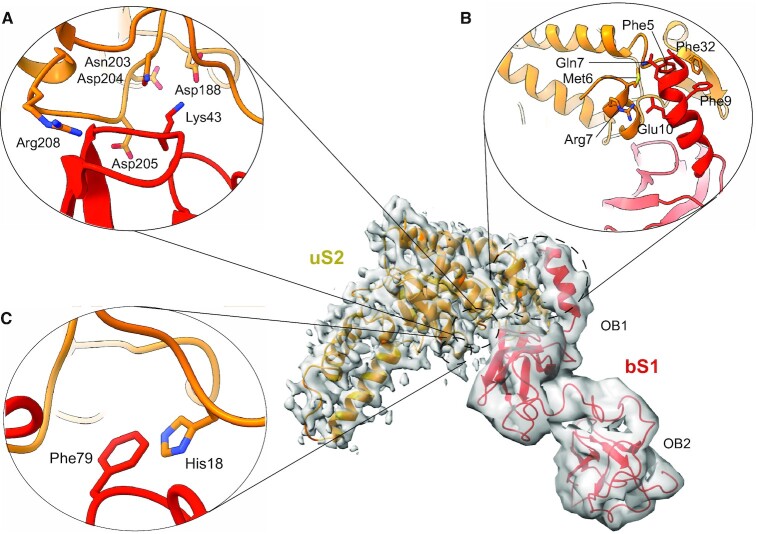
bS1 interaction with uS2. (**A**) Interaction between loop 2 in bS1 (red) and the Zn^2+^-binding pocket of uS2 (orange). (**B**) Interactions between uS2 (orange) and the OB1 N-terminal helix of bS1 (red). (**C**) Close-up of the novel stacking between bS1 Phe79 and uS2 His18 residues.

In agreement with previous studies (19,20), we found that the bS1 N-terminal domain binds to the cleft between the head and the platform, in a zone consisting of uS2, bS18 and bS21 residues as well as helix h26 and the 3′ end of the 16S rRNA. The volume obtained from single particle analysis shows not only the bS21 OB1 and OB2 domains, but also the mRNA SD portion paired to the 16S aSD (Figure 1B). Unfortunately, no other densities are present for the 5′ UTR sequence upstream from these six nucleotides, nor for the other bS21 domains (OB3–OB6). The major anchoring point for bS1 on the *E. coli* ribosome is the uS2 protein (53,54). The OB1 N-terminal helix is the most well-resolved element, confirming its conformational stability and its role in binding the entire protein to the ribosomal platform. As already seen in other bS1 structural studies (19,20), the OB1 N-terminal helix binds the uS2 protein via a classical π-stacking between the Phe5 and Phe9 residues of bS1 and the Phe32 of uS2 (Figure 2A). In addition to these previously described interactions, however, we saw other electrostatic interactions: bS1, Gln7 and Glu10 interact with uS2, Met6 and Arg7, respectively, and this is probably important for the global stability of OB1 on the ribosome (Figure 2A). The extreme tip of bS1’s loop 2 packs on two uS2 α-helices, and this allows the insertion of Lys43 into a zinc-binding pocket formed by Asp188, Asn203, Asp204 and Asp205 (Figure 2B) (19,20). The uS2 Arg208 points in the opposite direction as the bS1 Asp39 residue, and the salt bridges described by Byrgazov *et al.* (19) were not observed in our structure. However, in a previously unseen interaction, the aromatic rings of bS1 Phe79 and uS2 His18 were involved in a classical π-stacking in our structure (Figure 2C), another novel interaction contributing to the overall stability of OB1 on the ribosome. This confirms the roles of both uS2 and bS1’s OB1 domain for ensuring the temporary stable interaction essential for bS1 to correctly position the mRNA. Taken together, these observations prove that it is not only Phe5, Phe9 and Lys43 residues, but also Gln7, Glu10 and Phe79 that participate in binding bS1 to uS2. Therefore, during the early steps of protein synthesis, it is the entire OB1 that stabilizes bS1 at the 30S surface, while the other domains remain inherently free and flexible.

### The bS21 protein is involved in bS1 stabilization on the ribosome

**Figure 3.**
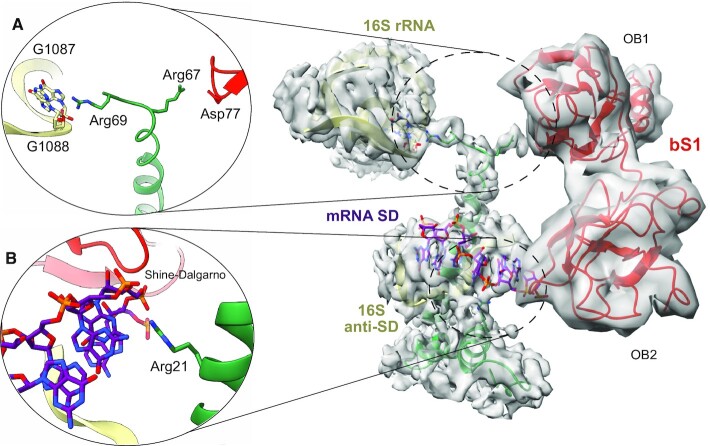
bS1 OB1 interactions with bS21. (**A**) The ribosomal protein bS21 shows a novel hinge activity between the 16S rRNA (khaki) and OB1 (red), the first N-terminal domain of bS1. (**B**) Close-up showing the interaction between bS21 (aquamarine) and the SD portion of mRNA (purple).

The protein bS21 is the smallest (70 amino acids) and most basic protein in the small ribosomal (30S) subunit of bacteria (55,56). It assumes a particular conformation in the cleft between the head and the platform of 30S, thus participating in the formation of the mRNA exit channel (56). Together with bS1, bS21 is required for the initial steps of protein synthesis, favouring the base pairing between 16S rRNA and the SD portion of mRNA (56,57). In agreement with the studies cited here, we saw that bS21 participates in the base pairing between 16S and mRNA by directly interacting with the SD sequence via two arginines (Arg17 and Arg21) positioned at the protein’s N-terminal region (Figure 3B). The C-terminal end of bS21 protrudes towards the 30S platform in a previously unseen hinge function between bS1 OB1 and the 16S rRNA (Figure 3A). Indeed, our structure reveals that the Arg67 and Arg69 residues in bS21 point in two opposite directions, with Arg67 interacting with bS1 Gly78 and Arg69 contacting nucleotide G1099 of the 16S rRNA (Figure 3A). This conformation allows bS21 to act as a second anchoring point for bS1, certainly strengthening its binding with the ribosome. These observations confirm the role of bS21 in translation initiation not only via its promotion of base pairing between the SD and aSD sequences, but also by acting as a bridge to reinforce the binding of the bS1 OB1 to 16S rRNA.

### OB2 stabilizes the base pairing between the SD and aSD sequences

**Figure 4.**
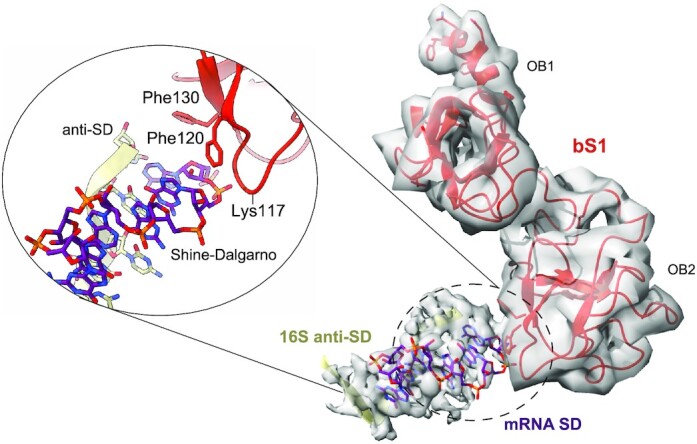
bS1 OB2 interactions with the mRNA exit channel. Close-up showing the interactions between the bS1 OB2 pocket (red) and the SD portion of the mRNA (purple).

Our new data also allowed us to reconstruct the OB2 domain, yielding fundamental insights into how bS1 interacts with its partners. The OB2 domain flanks the mRNA exit channel, in close vicinity to the SD–aSD helix (Figure 4). The α-helix connecting OB1 to OB2 is mostly unfolded, which explains the partial rotation of OB2 as well as why the domain appears in a totally different conformation than in previously described structures (Supplementary Figure S4) (16,20,58). Here, the aromatic rings of two phenylalanine residues, Phe120 and Phe130, form a patch welcoming the first nucleotides of the mRNA SD segment (Figure 4). Together with Lys117, these residues could form a binding pocket that captures the mRNA in a pincer movement, suggesting that OB2 also plays a role in stabilizing the SD–aSD interaction. This particular conformation corroborates previous observations that bS1 requires a particular orientation of its first three OB domains to bind RNA molecules and unfold structured messenger RNA, and that deletion of OB1 and OB2 not only decreases the binding rates of bS1 to the ribosome, but also damages its ability to interact with mRNA (19,24). Indeed, the new positioning of OB2 and the presence of such a binding pocket might provide an explanation for why the bS1 N-terminal domains are needed not only to anchor the protein to the ribosome, but also for stabilizing and correctly placing the mRNAs there, and thus for allowing the 30S initiation complex to be formed.

On the OB2 domain, the conformation of the pocket suggests that it can work as a groove to stabilize mRNA while the base pairing with the SD is being formed. Unlike other structural studies of bS1 (16,19,20) stating that most of the interactions around the mRNA exit channel are with OB3, we actually found that after the formation of the 70S EC complex, bS1 places its OB2 domain into the cleft of the 30S subunit. This suggests that OB2 is involved in correctly positioning the mRNA into the 30S late initiation complex as well as in stabilizing the SD–aSD base pairing. With the exception of bS18 and uS2, no interactions between OB2 and other ribosomal proteins were observed.

## DISCUSSION

Translation initiation is the rate-limiting step in protein synthesis. It depends mostly on the presence of recruitment signals in the 5′ UTR leader region, as well as several protein factors, which assist in forming the 70S EC (49,59). The ribosomal protein bS1 is one of these agents, helping mRNAs to reach the ribosome and then ensuring their proper accommodation into the decoding channel even in the absence of a 5′ UTR or proper SD sequence (24,59). Two distinctive features of bS1 are its flexibility and dynamicity, but this means that we still lack a full-length atomic description of the protein. The detailed model we present here consists of the first two N-terminal domains of bS1 in complex with a 70S EC ribosome charged with an mRNA and a tRNA^fMet^. The in-depth classification of the particles in our cryo-EM dataset uncovered a completely unique conformational rearrangement of bS1 that had not been observed previously. Due to the presence of the SD–aSD pairing, our structure reveals a more detailed interaction between the two first domains of bS1 and the 70S EC. The other four domains of the protein are not stable enough to be modelled, and their description may require the presence of a longer and structured 5′ UTR region.

The N-terminal segment of the OB1 domain is folded into an α-helix that mediates its interaction with uS2 via a well-conserved and well-described π-stacking involving bS1 Phe5 to Phe9 and uS2 Phe32 residues (Figure 2A) (19,20). The high stability of the N-terminal portion ensured by this hydrophobic interaction is also reinforced by electrostatic interactions described here for the first time as a result of improved resolution. In particular, bS1 Gln7 and Glu10 interact with uS2 Met6 and Arg7, respectively (Figure 2A). In perfect agreement with previous findings, the bS1 Lys43 residue found on the extremity of loop 2 is inserted into the uS2 Zn^2+^-binding pocket (Figure 2B) (19,20). Based on previous knowledge of the Zn^2+^-binding function of uS2 and on crystallographic results from structural studies, we are able here to confirm the positioning of Lys43 within this pocket formed by uS2 Asp188, Asn203, Asp204 and Asp205 (19,60). The residues involved in forming the pocket are conserved among different species, underlining the importance of the Zn^2+^ ion for stabilizing the uS2 structure and correctly positioning bS1 (19). The most novel element that completes the ribosome interaction picture is the presence of a stacking between bS1 Phe79 and uS2 His18 (Figure 2C). If we look at the sequence alignment of the uS2 proteins (Pfam PF00318, 68 244 sequences), the His18 is conserved 100% of the time. The situation is more complex because bS1 F79 is in a loop. However, there is a clear prevalence for phenylalanine (F 24%) or acidic residues (E 20% and D 10%) at this position in the 49 sequences retrieved from UniProtKB. These residues could all interact with uS2 His19. Moreover, we cannot exclude that adjacent residues can also play a role in the interaction. Taken together, these results support the idea that this interaction is certain to further stabilize bS1 at the surface of the ribosome.

In addition, our study shows that an important role in the ribosome–bS1 interaction is also played by the protein bS21, whose ability to interact with bS1 itself was previously observed (16). Our high-resolution structure allows for a clearer description of these interactions, and clarifies the role of bS21 during translation initiation. Acting as a three-way bridge, the C-terminal portion of bS21 mediates the interaction between the bS1 OB1 domain and 16S rRNA via two arginines (Figure 3A). The N-terminal extremity of bS21 also makes contact with the 3′ end of the 16S rRNA, creating an elegant three-point contact system that further anchors bS1 to the ribosomal platform (Figure 3B). In the course of our analysis, we also uncovered a possible binding pocket in the OB2 domain (Figure 4). This previously unseen binding site that could be formed by Lys117, Phe120 and Phe130 surrounds the first nucleotides of the SD sequence of the mRNA (Figure 4). In comparing our model with that of Loveland and Korostelev (20), we see that the residues involved in forming this pocket point in the opposite direction (Supplementary Figure S5). We are therefore describing a different bS1 conformation in which the OB2 domain rotates, thanks to a partial misfolding of the helix connecting OB1 to OB2, thus further emphasizing the protein’s intrinsic dynamicity (Supplementary Figure S4). The partial loss of the α-helical conformation allows the protein to position the OB2 domain residues exactly upstream from the mRNA SD element. Interestingly, a ConSurf (61) analysis of bS1 (Supplementary Figure S4) using a sample of 150 sequences (the homologues) reveals that Phe120, Phe130 and Lys117 present high conservation scores. This analysis reinforces the idea that these residues have a specific function, as they recur systematically in different species. When comparing the atomic model derived from this study with the one obtained by NMR (62), the overall organization of the OB1 and OB2 domains is well conserved. The secondary structure elements, typical of the OB-fold domain, are present and conserved at the same positions. The main difference is noticed when comparing with OB2 (Supplementary Figure S6A and D). The conformation of the ribosome-binding domain is in our case more ‘open’, with an outward shift of the three β-strands. However, the portion formed by the flexible loop is much more pronounced in the NMR model. This difference reflects the dynamic nature of this element. When comparing with OB2 domains from the two other cryo-EM structures (20,57), once again the major difference is due to the position of the flexible loop of OB2 (Supplementary Figure 6B, C, E and F). Finally, the only questionable element is the complete absence of the α-helix within our model, an element that normally serves as a connection between OB1 and OB2. However, the absence of this secondary structure element is due to the rotation of OB2, necessary to position the hydrophobic pocket (F120–F130) at the level of the SD–aSD interaction. The partial loss of this element is therefore necessary for this domain to perform such a particular new function.

Until now, it was thought that the first two OB domains of bS1 were involved only in anchoring the protein to the ribosomal platform, with the other domains intervening in the capture, binding and unfolding of leaderless or structured mRNAs. However, our data show that OB2 also interacts with the 5′ end of the SD through the formation of a characteristic pocket in which the mRNA nucleotides are accommodated. This new role for OB2 is supported by 3D density maps published by Loveland and Korostelev (20) showing some bS1 conformations with OB-fold domains checking the mRNA exit channel, with the OB2 near the 5′ end of the mRNA. During translation initiation, this pocket could act as clamp to stabilize Watson–Crick SD–aSD interactions. Our atomic model presented features of a 70S subunit in the EC state wherein the association of the two subunits around a tRNA^fMet^ produces a ribosome that is ready to translate the mRNA. At this stage, bS1 still binds both the ribosome and the initial region of the mRNA. This particular conformation of the second domain of bS1 can be explained by the topological rearrangement occurring after RNA molecules are bound, which in turn causes a global reorganization of the protein on the ribosome via other interactions involving OB1, OB2, uS2 and bS18, as described previously (18,19). Collectively, these interactions facilitate the correct positioning of the mRNA, and might also facilitate the transition from the 30S initiation complex to the 70S EC.

To conclude, the results presented here broaden the collective knowledge of the functionality of one of the most flexible ribosomal proteins. They emphasize the functional versatility of the OB2 domain, and highlight several new interactions essential for the stability of bS1 during translation initiation. Together with the contacts previously described in other studies (19,20), our results emphasize the importance of new accessory interactions between bS1 and uS2. We also demonstrate that bS21 is essential in the binding between bS1 and the 30S small ribosomal subunit, acting as a three-way clamp indirectly connecting OB1 to the 16S. In particular, we clarify the role of this protein’s OB2 domain by describing a previously unseen conformation allowing it to interact directly with the SD portion of mRNA. In this way, the OB2 domain actively participates in the formation of the SD–aSD pairing, giving life to the 30S initiation complex.

## DATA AVAILABILITY

The electron density maps and structure models are deposited in the EMDB and PDB under the following accession codes, respectively: EMD-15116 and 8A3L.

## Supplementary Material

gkad126_Supplemental_File
