# Supplementary material for: Structural insights into the binding of bS1 to the ribosome
Source: Nucleic Acids Res. 2023 Feb 25;51(7):3410–9. doi: 10.1093/nar/gkad126 (PMC10123108; doi:10.1093/nar/gkad126)
Supplement: gkad126_Supplemental_File [file gkad126_supplemental_file.docx]

**SUPPLEMENTARY INFORMATION**


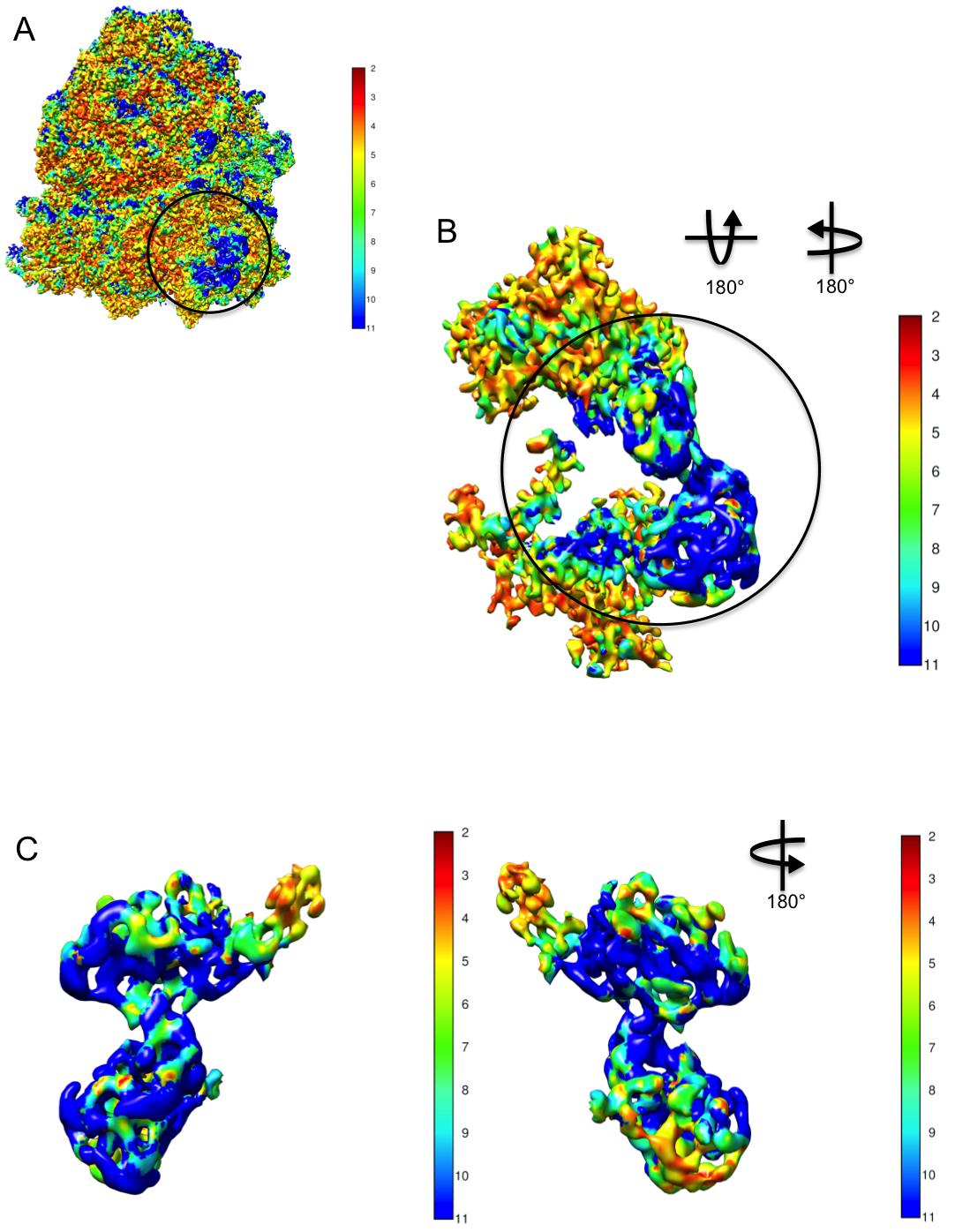


***Supplementary Figure 1: cryo-EM map of the ribosome-bound bS1 protein.*** *A – Local-resolution map of the translating ribosome in complex with bS1 N-terminal domains. B – Focus on the bS1 binding site at the mRNA exit channel. C – Local-resolution map of bS1 N-terminal domains for the side that faces the external surface (left) and that which faces the ribosome (right).*

***Supplementary Figure 2: Image analysis protocol.*** *Workflow adopted during the image analysis in order to obtain the final cryo-EM map*

*
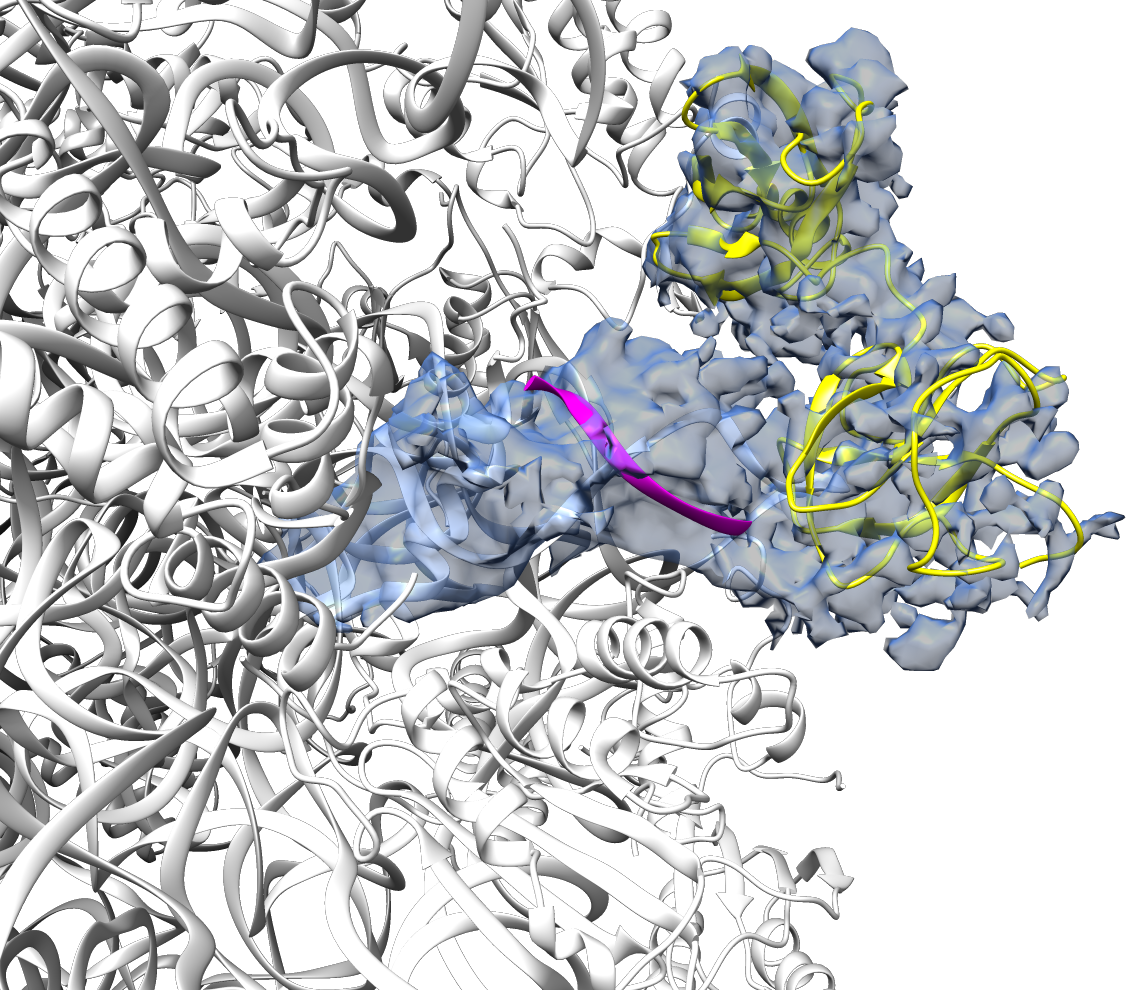
*

***Supplementary Figure 3****:* Cryo-EM density map of bS1 on a stalled ribosome. For clarity only the portions of the map corresponding to bS1 and SD-antiSD are shown. bS1 (OB1 and OB2) is in yellow, the mRNA (sequence 5’-AGGAGGUGAGGUUUU-3’) is in pink and 70S in light grey.


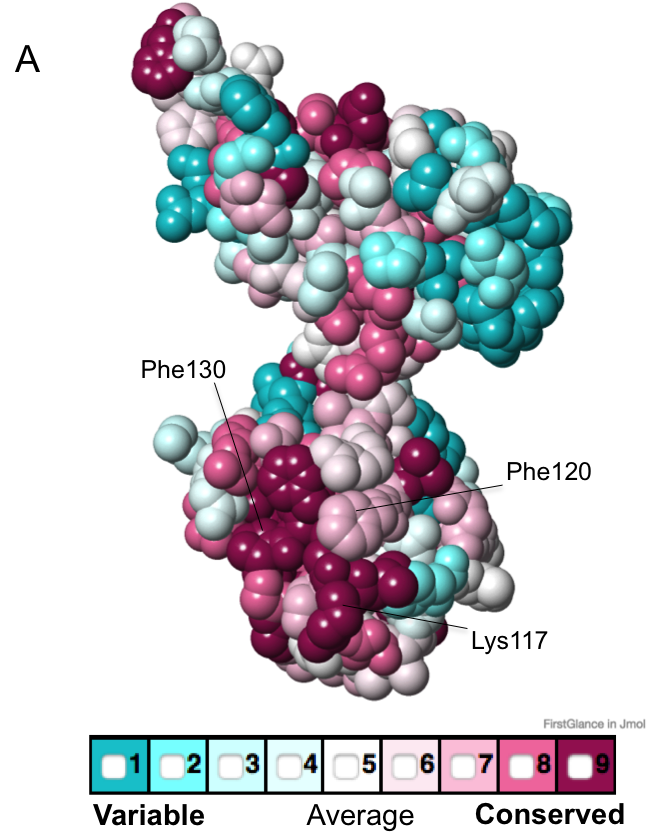


***Supplementary Figure 4: Sequence analysis between bS1 and its homologues.*** *Analysis of the conservation scores of the residues forming the first two N-terminal domains of bS1. The scores are calculated based on the sequence alignments of 150 bS1 homologues, and range from low (blue) to high (purple).*


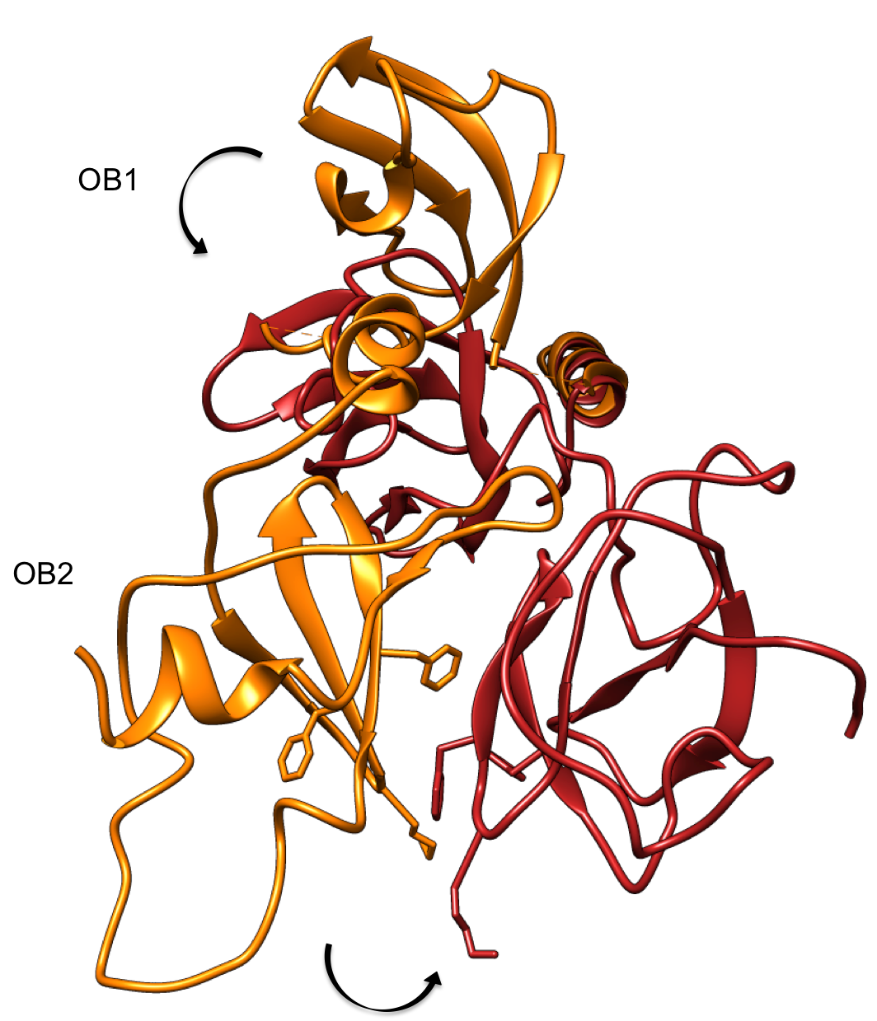


***Supplementary Figure 5: Atomic model comparison between our model and a previous cryo-EM bS1 structure.*** *Comparison between the new conformation described in our bS1 model (red) and the conformation reported in previous structural studies (20) - PDB file 6BU8, orange.*

***Supplementary Figure 6: Comparison of the atomic model of OB2 derived from this study with the other published structures.*** *Comparison of the current model with the one obtained by NMR (****A****) and (****D****); cryo-EM of hibernating 100S ribosome (****B****) and (****E****); cryo-EM of 70S ribosome (****C****) and (****F****).*
